# Effectiveness of an online versus face-to-face psychodynamic counselling intervention for university students before and during the COVID-19 period

**DOI:** 10.1186/s40359-022-00742-7

**Published:** 2022-02-22

**Authors:** Elena Ierardi, Marta Bottini, Cristina Riva Crugnola

**Affiliations:** Department of Psychology, University of Milano-Bicocca, Piazza dell’Ateneo Nuovo 1, 20126 Milan, Italy

**Keywords:** Psychodynamic university counselling, Psychopathological problems, Online university counselling intervention, Life satisfaction, Attachment styles

## Abstract

**Background:**

The COVID-19 pandemic has increased online counselling interventions, including those aimed at university students. The principal aim of the study is to evaluate the effectiveness of the online counselling intervention during the COVID-19 pandemic, also with regards to the effectiveness of the face-to-face intervention.

**Methods:**

34 students (Mean age = 23.74; Female = 27) who requested online university counselling during COVID-19 have been compared with 81 (Mean age = 22.8; Female = 60) students who requested university face-to-face counselling before the pandemic. The psychopathological problems were assessed with the Symptom Checklist 90 Revised, attachment styles with the Attachment Style Questionnaire, adverse childhood experiences with Adverse Childhood Experiences Questionnaire, and life satisfaction with the Life Satisfaction Scale.

**Results:**

At the pre-intervention phase, psychological distress was similar in both groups with no differences in the General Severity Index of the SCL-90 R, and there were no significant differences for secure/insecure attachment, adverse childhood experiences, and life satisfaction. The online counselling intervention during the pandemic was effective in reducing psychological distress scales as depression (*p* = .008), obsessive–compulsive (*p* = .008), interpersonal sensitivity (*p* = .005), and anxiety (*p* = .011), and in the total scale of the SCL-90 R (*p* = .017). The face-to-face counselling intervention was effective in reducing psychological distress in all subscales and in the total scale of the SCL-90 R (*p* = .000) and in increasing the level of life satisfaction (*p* = .023). Attachment style did not moderate the effectiveness of the online and face-to-face interventions.

**Conclusions:**

Students seeking counselling, both before and during the pandemic, show similar levels of psychological distress. The online counselling intervention was almost as effective as face-to-face counselling intervention with respect to psychological distress; it was not effective in increasing life satisfaction.

## Background

The consequences of the spread of COVID-19 and of self-isolation and quarantine measures have led to radical changes in people’s daily lives, such as loneliness, high levels of depression, anxiety, alcohol abuse, drug use, self-harm, domestic violence, sleep problems, and suicidal behaviors [1, 2]. In fact, many studies have shown a high increase in psychological problems shortly after the declaration of the state of emergency [3–5] with an increase of 20–30% of depression and anxiety compared to the pre-pandemic period [6].

Also, studies have identified the impact of COVID-19 on university students’ mental health.

During this period, the students suffered from anxiety and depression as well as social dysfunctions [6, 7], stress, concentration disorders, and psychosomatization [8]. At the same time, students reported concerns about the lack of social activities and their future career opportunities, especially those who were closer to graduation [9].

To face the state of emergency and the growing psychological problems in the pandemic period, the services that provide psychological help have had to change place and space, transforming their intervention from face-to-face, to online intervention [10]. Online psychological interventions were present even before the pandemic emergency; with the rapid development of technologies, in fact, starting from the twenty-first century, there has been an increasing possibility of carrying out psychological counselling online [11, 12].

Considering the importance of mental health interventions aimed to young adult [13], it is extremely useful to examine whether such online counselling interventions for university students are effective in improving subjective well-being as well as face-to-face counselling interventions do. In fact, counselling has an important role in supporting students with psychological problems, promoting a reduction of psychological distress and an increase in psychological resilience and academic performance [14, 15].

Studies done before the pandemic period showed that online counselling can be as effective as face-to-face counselling for general population [16, 17]. In this regard, Mullin et al. [18] showed that an online wellbeing program reduced the anxious and depressive symptoms compared with the control group at post-treatment and at a three-month follow-up. Other studies have shown that online counselling is also effective for people using substances [19] or showing psychological distress [20]. Furthermore, studies show that patients have high levels of satisfaction after having completed an online counselling process [21] and are able to express their discomfort as well as face-to-face counselling interventions [22]. A study carried out during the pandemic [23] showed the effectiveness of single online psychological counselling session in reducing anxiety and negative affect.

With regards to university students, some systematic reviews, carried out before the pandemic, showed that mental health interventions based on online with computer and phone interviews appear effective, particularly in improving anxiety symptoms [24, 25]. However, studies comparing online counselling interventions with face-to-face interventions for university students found mixed results. Among these, Chan [26] compared in a group of teens and young adults the effectiveness of the counselling intervention provided online, offline and mixed (online plus face-to-face), showing that the most effective in terms of youth’s quality of life and sense of well-being was from mixed counselling and that the online counselling was more effective than face-to-face. Zeren et al. [27], comparing the effectiveness of online individual counselling, face-to-face counselling and a control group in undergraduate students, have not found significant differences among the types of intervention, highlighting that online counselling can be as effective as face-to-face counselling in improving satisfaction. However, only in the group with face-to-face intervention there was a significant increase in positive affect and a decrease in negative affect.

No study has evaluated the effectiveness of online interventions, comparing it with the face-to-face counselling interventions during the pandemic period; furthermore, no one has compared different approaches to counselling intervention aimed at university students, such as psychodynamic or cognitive-behavioral one. Only the study by Savarese et al. [8] qualitatively assessed online counselling for university students during pandemic period of COVID-19, suggesting that online counselling services for university students in times of emergency helped students to increase their resilience skills and is useful to identify psychological problems.

It is clear from above that the analysis of the effectiveness of online counselling interventions is an important field of research in which studies and empirical evidence are needed.

Quality of attachment styles might affect the effectiveness of counselling interventions in improving mental health. Several researchers have pointed out that insecure attachment can be considered a general factor of vulnerability with respect to psychopathology. For example, in the adult population both anxious and avoidant attachment styles are associated with depression, anxiety, obsessive–compulsive disorder, and externalizing pathologies [28–30]. A study by Riva Crugnola et al. [31] also showed correlations between psychopathological problems and insecure attachment in a group of university students. Other studies [32, 33] found that anxious attachment predicted negative mental health outcomes during COVID-19 period, leading higher levels of depression and anxiety.

### Approach of the study

The main aim of the study is to evaluate the effectiveness of a psychodynamic counselling intervention carried out online through video sessions with university students during the COVID-19 pandemic and to compare its effectiveness with the face-to-face psychodynamic counselling intervention, carried out before the pandemic.

The first aim is to assess psychological distress before the pandemic and during the pandemic in university students requiring university counselling. It is hypothesized that students during the pandemic had an increase in psychopathological distress compared to students who had requested counselling prior to the pandemic, based on recent literature [7]. The second exploratory aim—as aren’t enough studies on the subject—is to evaluate the effectiveness of the online intervention and compare its effectiveness with the face-to-face one. The third aim, also at an exploratory level, is to assess whether the attachment style can be a moderator of the effectiveness of the online counselling intervention and of the face-to-face counselling intervention.

## Method

### Participants and procedure

The participants in our study were students of the University of Milano-Bicocca who referred themselves to the Counselling Service. The first group of 34 university students requested and carried out online counselling during the emergency situation caused by the COVID-19 from January 2020 to July 2021 and completed the battery of questionnaires both before the intervention (T0-baseline) and after the intervention (T1-after 4 counselling sessions). The second comparison group of 81 university students required counselling between January 2016 and December 2019 and carried out the intervention face-to-face. The socio-demographic characteristics of the participants are reported in Table 1.

**Table 1:** Socio-demographic profile of two groups

	Online counselling group (N = 34)	Face-to-face counselling group (N = 81)	*p*
Age (Mean; SD)	23.74; 3.25	22.8; 2.56	n.s
Sex			
Female	27 (79.4%)	60 (74%)	n.s
Male	7 (20.6%)	21 (26%)	
Marital status			
Single	32 (94.2%)	78 (96.2%)	n.s
Married/living with a partner	2 (5.8%)	3 (3.8%)	
Living arrangement			
With a partner	4 (11.8%)	4 (5%)	n.s
With parents	24 (70.5%)	60 (74%)	
Alone	0 (0%)	3 (3.7%)	
With friends	4 (11.8%)	11 (13.6%)	
Other	2 (5.9%)	3 (3.7%)	
Occupation			
Job	9 (26.4%)	17 (21%)	n.s
No job	25 (73.6%)	64 (79%)	
Parenthood			
With children	0 (0%)	1 (1.2%)	n.s
Without children	34 (100%)	80 (98.8%)	
Department			
Psychology	14 (41.1%)	45 (55.6%)	n.s
Human sciences	14 (41.1%)	26 (32%)	
Laws and economics	4 (11.7%)	10 (12.4%)	
Mathematical and physical sciences	2 (6.1%)	0 (0%)	
Degree’s course			
Bachelor students	17 (50%)	35 (43.2%)	n.s
Master students	9 (26.4%)	31 (38.2%)	
Supplementary year students	8 (23.6%)	4 (18.6%)	

Number of subjects (N)

The data collection procedure takes place entirely online, after the acquisition of written informed consent, and students who voluntarily decide to participate fill in batteries of questionnaires administered at T0 and readministered at T1. All procedure involving human participants were performed in accordance with the ethical standards of the institutional and/or national research committee and with the 1964 Helsinki Declaration and its later amendments or comparable ethical standards. The ethics committee of the University of Milano-Bicocca approved this research project.

### Intervention approach

The intervention has been conducted at the University of Milano-Bicocca Counselling Service and is open to all students of the campus. It’s a psychodynamic intervention which offers students a brief consultation intervention composed of four meetings, conducted *vis-à-vis* [15, 34, 35]. The intervention starts with the student referring him/herself for counselling and is free for all students enrolled at the university. The intervention approach is focused on identifying the main problems that hinder the student's developmental process of identity and relational consolidation [36].

The sessions function as a transitional space, protected from family and academic pressures. The primary objective of the intervention is, therefore, to make an alliance with the student, in order to create a “safe” relational context [37]. This should improve his/her capacity to address difficulties and distress encountered in the academic life or connected to developmental crises that are typical of emerging adulthood [38, 39].

From this perspective, the intervention aims to make the student aware of the conflictual nodes, underlying the request for consultation, as to increase his/her capacity to reflect on his/her mental and emotional states and on those of other people (relatives, classmates, partners, lecturers). Intervention in this way can be useful both to promote processes of elaboration and mentalization in students, allowing for initial processing of these problems [40] and to mobilize their resources which may be used to set in motion decision-making and responsibility assumption processes.

The team is composed of psychologists trained in psychodynamic psychotherapy and in psychodynamic counselling. The psychologists are supervised in group regularly every fifteen days by the service coordinator who is a psychoanalyst. During the COVID-19 period, the intervention was carried out online through video sessions using the same approach adopted in face-to-face interviews.

### Measures

#### Psychopathological problems

Symptom Checklist 90 Revised (SCL-90 R) [41, 42] is a 90-item self-report questionnaire (rated on a 5-point Likert scale ranging from 0 “not at all”, to 4 “extremely”) that measures the perceived severity of psychopathological symptoms over the previous seven days. SLC-90 R includes 9 subscales: Somatization (SOM), Obsessiveness-Compulsivity (O–C), Interpersonal Sensitivity (I-S), Depression (DEP), Anxiety (ANX), Hostility (HOS), Phobic Anxiety (PHOB), Paranoid Ideation (PAR) and Psychoticism (PSY). The instrument also has three global indexes—Global Severity Index (GSI), Positive Symptoms Total (PST) and Positive Symptoms Distress Index (PSDI). In this study, we used the Global Severity Index as a global index. All the subscales and the global index showed good reliability in this study (.76 < α < .96).

#### Life Satisfaction

Life Satisfaction scale is a 9-point Likert scale (from 1 “very dissatisfied” to 9 “very satisfied”). Students are asked to indicate their general life satisfaction and satisfaction in relation to Study, Work, Friends, Family, Romantic Relationships, and Free time. The items of this scale were taken from the Italian version of the Response Evaluation Measure-71 [36]. In our study we used the general life satisfaction level scale, adding one item on study satisfaction that was not present in the original measure. Even though no data is available on the validity of this measure, we relied on the high face validity of these items.

#### Attachment Style

Attachment Style Questionnaire (ASQ) [43, 44] is a 40-item self-report scale (evaluated on a 6-point Likert scale ranging from 1 "Totally disagree" to 6 "Totally agree") which yields five factor scores. One factor (Confidence in self and others) is related to secure attachment, whereas each of the other four scales (Discomfort with Closeness, Relationships as Secondary, Need for Approval, and Preoccupation with Relationships) represents a particular aspect of insecure attachment. We administered the ASQ only at T0.

The ASQ scales were grouped together in order to highlight differences with respect to the two types of insecure attachment. As indicated by Fossati et al. [45] through the four scales which measure insecure attachment it is possible to identify the dimensions of insecurity: Avoidance and Anxiety. Following Monteleone et al. [46], two new scales were created relating to insecure attachment: Avoidant Attachment which is the average of the scores of the Discomfort with Closeness and Relationships as Secondary scales and Anxious Attachment which is the average of the scores of the Preoccupation with Relationships and Need for Approval scales. The scales showed good reliability in this study (.74 < α < .77).

#### Adverse Childhood Experiences

Adverse Childhood Experiences Questionnaire (ACE-q) [47] is a self-report questionnaire consisting of 10 items, aimed at investigating the adverse experiences that the subject lived before the age of 18, such as physical, sexual, psychological abuse, losses, separation and neglect. For each question, the subject can assign a score of 0 (no—if he/she has not experienced the adverse experience in question) or 1 (yes—if he/she has experienced the adverse experience mentioned). The final score is calculated by adding up the answers. Based on the literature, there is a consistent presence of adverse experiences in the history of the subject with a cut-off greater than or equal to 4 [48]. We administered the ACE-q only at T0.

## Data analysis

We used SPSS Statistic 27.0 package for all analyses. First, in order to understand if the two groups are similar, we analyzed the demographic variables through Chi-square test for categorial variables and t-tests for continuous variables. We then analyzed any differences between the two groups in psychopathological distress, attachment style, life satisfaction, and adverse experiences at T0 through the t-test. We conducted dependent t test for each group to evaluate the effectiveness of the online and face-to-face interventions on the SCL-90 R and level of life satisfaction. Moreover, we conducted a more detailed analysis with repeated measures univariate analysis to evaluate whether there were differences in effectiveness between online and face-to-face intervention in reducing the level of distress and in increasing the level of life satisfaction. To analyse the changes groupXtime interactions, a two-way mixed design repeated measures analysis of variance was performed.

We also evaluated attachment style as a possible moderator using the MEMORE (Mediation and Moderation for Repeated Measures) procedure, a plug in for SPSS to conduct moderation analysis with repeated measures designs [49]. Cohen’s d and partial eta squared was considered as the effect size. The effect size values considered were 0.10 = small, 0.25 = medium, and 0.40 = large.

## Results

### Preliminary analysis

Preliminary analyses with chi-square and t-test did not show significant differences between online counselling group and face-to-face counselling group on socio-demographics characteristics (see Table 1).

### Differences between the two groups

We used t-test to analyse the differences between the online counselling group and face-to-face counselling group on psychopathological distress, life satisfaction, attachment style, and adverse childhood experiences (see Table 2).

**Table 2:** Difference between groups on SCL-90 R, ASQ, life satisfaction, and ACE

	Online counselling Group (N = 34) *M* (*DS*)	Face-to-face counselling Group (N = 81) *M* (*DS*)	t	*p*
SCL-90 R				
Somatization	.78 (.61)	.82 (.79)	− .26	.79
Obsessive–compulsive	1.30 (.84)	1.54 (.80)	− 1.43	.15
Interpersonal sensitivity	.95 (.70)	1.18 (.75)	− 1.50	.13
Depression	1.38 (.77)	1.62 (.83)	− 1.46	.14
Anxiety	1.06 (.67)	1.15 (.81)	− .54	.59
Anger-hostility	.69 (.57)	.90 (.85)	− 1.52	.13
Phobic anxiety	.48 (.65)	.51 (.68)	− .19	.84
Paranoid ideation	.71 (.66)	1.02 (.74)	− 2.06	.04*
Psychoticism	.57 (.64)	.80 (.60)	− 1.89	.06
GSI	.95 (.56)	1.12 (.60)	− 1.44	.15
ASQ				
Confidence	29.43 (5.76)	28.16 (5.57)	1.08	.28
Discomfort with closeness	33.06 (6.68)	39.06 (9.52)	− 3.78	.000***
Relationships as secondary	14.09 (4.21)	15.57 (6.23)	− 1.45	.15
Need for approval	22.46 (6.70)	25.51 (6.89)	− 2.12	.036**
Preoccupation with relationship	29.65 (6.61)	31.85 (7.12)	− 1.50	.13
Avoidant attachment	23.57 (4.84)	27.46 (7.16)	− 3.32	.001***
Anxoius attachment	26.06 (5.81)	28.74 (5.86)	− 2.19	.030**
Life satisfaction	5.50 (2.06)	4.88 (2.07)	1.37	.17
ACE	1.19 (1.37)	1.59 (1.38)	− 1.17	.24

M(DS), mean and standard deviation; *p*, level of significance

^*^p < .05, **p < .01, *** *p* < .000

Regarding SCL-90 R, no significant differences emerge with regards to the total scale and subscales except for the Paranoid Ideation subscale which results in having a higher score in the face-to-face counselling group. The two groups were compared through the Chi-square also in respect to the distribution of global psychopathological distress calculated through cut-off values of SCL-90 R GSI scores reported in the manual [50]. The Chi-square test showed no significant differences (X^2^ = 2.86; *p* = .23). In the online counselling group, 58% were in the non-clinical range, 23% in the subclinical and 19% in the clinical range. In the face-to-face intervention group, 41% were in the non-clinical group, 29% in the subclinical and 28% in the clinical group.

To analyze the attachment style in a dichotomous way security versus insecurity, we used the ASQ Confidence scale as indicator [45]: scores below the 25^th^ percentile were considered as an indicator of insecure attachment, whereas scores above the 25^th^ percentile were considered as secure attachment. The Chi-Square test indicated no significant differences in the distribution of secure/insecure attachment between the two groups (X^2^ = .80; *p* = .37). Instead, statistically significant differences emerge in the scales. Face-to-face intervention group students had higher score on Avoidant and Anxious Attachment scales, Discomfort with Closeness, and Need for Approval.

Finally, there are no significant differences between the two groups in terms of life satisfaction and in the amount of adverse experiences lived.

### Effectiveness of online counselling intervention

We examined the effectiveness of the online counselling intervention in online counselling group students on psychopathological problems and general life satisfaction level at T0 and T1 (see Table 3).

**Table 3:** Effectiveness of online counselling intervention

	T0 *M* (*DS*)	T1 *M* (*DS*)	t	*p*	d
SCL-90R					
Somatization	.79 (.61)	.69 (.57)	1.15	.25	
Obsessive–compulsive	1.31 (.84)	1.05 (.61)	2.69	.011*	.46
Interpersonal sensitivity	.96 (.70)	.66 (.48)	2.97	.005**	.51
Depression	1.38 (.77)	1.11 (.69)	2.82	.008**	.48
Anxiety	1.07 (.67)	.79 (.50)	2.80	.008**	.48
Anger-hostility	.69 (.57)	.58 (.62)	1.66	.10	
Phobic anxiety	.49 (.66)	.41 (.42)	.91	.36	
Paranoid ideation	.71 (.66)	.81 (.73)	− 1.03	.30	
Psychoticism	.58 (.54)	.53 (.50)	.69	.49	
GSI	.95 (.57)	.79 (.49)	2.51	.017*	.12
Life satisfaction	5.48 (2.10)	5.81 (1.98)	.90	.28	

M (DS), mean and standard deviation; *p*, level of significance; d, effect size

^*^p < .05, **p < .01

Dependent t-test indicated that there was a significant decrease in psychopathological symptoms from T0 to T1 in relation both to the GSI scale of SCL-90 R and to the Obsessive–Compulsive, Interpersonal Sensitivity, Depression, and Anxiety subscales. There is no significant increase in the overall level of satisfaction from T0 to T1.

### Effectiveness of face-to-face intervention

Then, we evaluated the effectiveness of face-to-face counselling intervention carried out with the university students before the pandemic, estimating any differences in psychopathological distress and general life satisfaction level at T0 and T1 (see Table 4).

**Table 4:** Effectiveness of face-to-face counselling intervention

	T0 *M* (*DS*)	T1 *M* (*DS*)	t	*p*	d
SCL-90 R					
Somatization	.83 (.78)	.59 (.64)	4.15	.000***	.46
Obsessive–compulsive	1.54 (.80)	1.22 (.79)	4.78	.000***	.53
Interpersonal sensitivity	1.18 (.75)	1.01 (.72)	2.67	.009**	.29
Depression	1.62 (.83)	1.22 (.83)	5.12	.000***	.57
Anxiety	1.15 (.81)	.87 (.71)	4.40	.000***	.48
Anger-hostility	.90 (.85)	.72 (.70)	2.21	.029*	.24
Phobic anxiety	.51 (.68)	.41 (.51)	1.74	.08	
Paranoid ideation	1.02 (.75)	.97 (.73)	.68	.49	
Psychoticism	.81 (.60)	.65 (.54)	3.22	.002**	.35
GSI	1.12 (.60)	.87 (.57)	5.37	.000***	.59
Life satisfaction	5.01 (2.00)	5.43 (1.66)	2.31	.023*	.26

M (DS), mean and standard deviation; *p*, level of significance; d, effect size

^*^p < .05, **p < .01, ***p < .000

Dependent t-test indicated that there was a significant decrease in psychopathological symptoms from T0 to T1 in relation both to the GSI scale of SCL-90 R and to the Somatization, Obsessive–Compulsive, Interpersonal Sensitivity, Depression, Anxiety, Anger Hostility, and Psychoticism subscales. There was a significant increase in the overall level of life satisfaction from T0 to T1.

### Effectiveness comparison of the two intervention modalities

To identify any differences in the effectiveness of the two interventions, repeated measures ANOVAs were conducted to test the timeXgroup interaction effects (see Table 5).

**Table 5:** Interaction effects timeXgroup

	F	*p*
SCL-90 R		
Somatization	2.02	.15
Obsessive–compulsive	.40	.52
Interpersonal sensitivity	.98	.32
Depression	.90	.34
Anxiety	.01	.91
Anger-hostility	.28	.59
Phobic anxiety	.07	.78
Paranoid ideation	1.26	.26
Psychoticism	1.32	.25
GSI	1.39	.24
Life satisfaction	.05	.81

*p* = level of significance

Results did not reveal any significant interaction effects between the group and the temporal dimension T0 and T1 for psychopathological distress and the level of life satisfaction.

### Moderation effect

We used MEMORE procedure to test the moderation effect of attachment style on the change of the psychopathological problems and on the level of life satisfaction in both groups. We used the secure/insecure attachment variable as a possible moderator (see above). The results showed that in the online counselling group students attachment style was not a significant moderator of the effectiveness of the intervention, both considering GSI Total scale, t(32) = .35, *p* = .72, and the level of life satisfaction, t(32) = − .93, *p* = .35.

Also, in the face-to-face intervention group students, attachment style was not a significant moderator of the effectiveness of the intervention, both considering GSI Total scale, t(81) = − .51, *p* = .60, and the level of life satisfaction, t(81) = − 1.24, *p* = .21.

## Discussion

The COVID-19 pandemic has led significant consequences on the mental health of university students. To face the emergency, online counselling interventions have increased, but however no study has evaluated the effectiveness of university online counselling during the pandemic period and no study has compared it with face-to-face counselling. Our study, therefore, filled a gap in a new research field, demonstrating the effectiveness of an online psychodynamic university counselling intervention during the COVID-19 pandemic. The online counselling intervention has proven to be able to reduce psychopathological distress in terms of depression, anxiety, obsessiveness-compulsiveness, and interpersonal sensitivity with a medium–high effect size as face-to-face counselling intervention. Even at the level of total psychopathological distress, both interventions were effective, showing the face-to-face intervention a large effect size and the online intervention a small effect size. A possible explanation for these results might be that a significant part of university students uses technologies in their daily life. Therefore, online counseling can be similar to face-to-face counseling in creating a good therapeutic alliance between psychologist and students based on empathy and listening [27, 51].

However, the face-to-face intervention has shown to have a greater impact than the online intervention on the discomfort and psychological well-being of students, also reducing the scores in anger, somatization and psychoticism subscales of SCL-90 R and promoting an increase in the level of general life satisfaction of students with a medium effect size. These results are in line with the study by Zeren et al. [27] which assessed pre-pandemic the effectiveness of online counselling for university students. They found that in general the online counselling intervention can be as effective as face-to-face counselling, but that only in the group with face-to-face intervention there was an improvement in positive affect.

A further relevant data emerged is that the quality of the attachment style is not a moderator of the effectiveness of the intervention neither for the face-to-face intervention nor for the online intervention in line with a previous study [15]. The intervention therefore has an effect in improving mental health both for university students with secure attachment styles and for university students with styles of attachment more at risk such as those insecure. This therefore shows the general effectiveness of the psychodynamic counselling model. It might be interesting, in a future study, to consider other moderators, including mentalization skills, personality structure, social support perception, and history of adverse childhood experiences.

Contrary to our initial hypothesis and the literature, university students who requested counselling during the pandemic did not show more pathological distress than students who required it before the pandemic. It should be noted, however, that usually students who request counselling intervention, have shown greater psychopathological symptoms than university students who did not request it [31, 52].

Moreover, students in the face-to-face intervention group had more avoidant and anxious insecure attachment styles than students of the online intervention. In this regard, it can be hypothesized that the pandemic may have also led students with secure attachment styles to undertake a counselling intervention for support in a moment of vulnerability. On the other hand, no significant differences emerged between the two groups regarding the presence of adverse experiences in their history.

The study has several limitations. Firstly, the small number of the group that carried out the online intervention limits the generalizability of the results. Secondly, the non-randomization of the two groups and the different periods in which the interventions—the online one during the pandemic period and the face-to-face one before the pandemic—were carried out could be another limit. Thirdly, we have not used a specific questionnaire to assess the psychological impact of the pandemic on university students. Finally, the effectiveness was assessed only at the end of the intervention, while in future studies it might be useful to evaluate whether the effectiveness is maintained over time with subsequent follow-up, e.g., after 6 months.

## Conclusions

The online counselling intervention for university students was overall effective in the reduction of general psychopathological distress and in many psychopathological dimensions. On the other hand, face-to-face interventions have proven to be more effective in reducing a wider spectrum of psychopathological problems and in increasing life satisfaction. The lower effectiveness of online interventions, especially regarding life satisfaction, could be linked to the pandemic situation itself and the resulting lockdown, which put a strain on the psychological well-being of the student population, inducing feelings of isolation and uncertainty. This—it can be hypothesized—has made it particularly difficult to increase satisfaction through short interventions. To take these aspects into account, it would be important to evaluate the effectiveness of online counselling in a non-emergency situation.

Given the overall effectiveness of online counselling in supporting students by reducing their psychological distress during the period of the COVID-19 pandemic, these data pave the way for the possibility of using it, together with face-to-face counselling, even in post pandemic times, e.g., to reach a larger number of students, such as off-site students or students who are afraid of social stigmatization in making use of mental health services.

## Data Availability

The datasets generated and/or analyzed during the current study are not publicly available but are available from the corresponding author on reasonable request.
